# Impact of SARS-CoV-2 Mutations on PCR Assay Sequence Alignment

**DOI:** 10.3389/fpubh.2022.889973

**Published:** 2022-04-28

**Authors:** Daniel Antonio Negrón, June Kang, Shane Mitchell, Mitchell Y. Holland, Stephen Wist, Jameson Voss, Lauren Brinkac, Katharine Jennings, Stephanie Guertin, Bruce G. Goodwin, Shanmuga Sozhamannan

**Affiliations:** ^1^Noblis, Inc., Reston, VA, United States; ^2^Joint Program Executive Office for Chemical, Biological, Radiological and Nuclear Defense (JPEO-CBRND), Joint Project Lead for CBRND Enabling Biotechnologies (JPL CBRND EB), Frederick, MD, United States; ^3^Noblis ESI, Chantilly, VA, United States; ^4^Logistics Management Institute, Tysons, VA, United States

**Keywords:** PCR, RT-PCR, COVID-19, SARS-CoV-2, diagnostics, pandemic, biosurveillance, signature

## Abstract

Real-time reverse transcription polymerase chain reaction (RT-PCR) assays are the most widely used molecular tests for the detection of SARS-CoV-2 and diagnosis of COVID-19 in clinical samples. PCR assays target unique genomic RNA regions to identify SARS-CoV-2 with high sensitivity and specificity. In general, assay development incorporates the whole genome sequences available at design time to be inclusive of all target species and exclusive of near neighbors. However, rapid accumulation of mutations in viral genomes during sustained growth in the population can result in signature erosion and assay failures, creating situational blind spots during a pandemic. In this study, we analyzed the signatures of 43 PCR assays distributed across the genome against over 1.6 million SARS-CoV-2 sequences. We present evidence of significant signature erosion emerging in just two assays due to mutations, while adequate sequence identity was preserved in the other 41 assays. Failure of more than one assay against a given variant sequence was rare and mostly occurred in the two assays noted to have signature erosion. Assays tended to be designed in regions with statistically higher mutations rates. *in silico* analyses over time can provide insights into mutation trends and alert users to the emergence of novel variants that are present in the population at low proportions before they become dominant. Such routine assessment can also potentially highlight false negatives in test samples that may be indicative of mutations having functional consequences in the form of vaccine and therapeutic failures. This study highlights the importance of whole genome sequencing and expanded real-time monitoring of diagnostic PCR assays during a pandemic.

## Introduction

Severe acute respiratory syndrome coronavirus 2 (SARS-CoV-2), like other Coronaviruses, is an enveloped virus with a linear, positive-sense single-stranded RNA genome. SARS-CoV-2 genomes range from 27 to 32 kb with an arrangement that is co-linear with other Coronaviruses. The genome is flanked by untranslated regions (UTRs) and protected by a 5′ 7-methylguanosine (m^7^G) cap and 3′ poly-A tail (1, 2). Genomic translation of ORF1ab yields polyproteins pp1a and pp1ab, which undergo autoproteolysis to yield non-structural (nsp) proteins involved in expression and replication (2). Transcription of the S (spike), E (envelope), M (membrane), and N (nucleocapsid) yields structural proteins for dissemination (2). Additional accessory protein genes include ORF 3a, 6, 7a, 7b, 8, and 9b (2, 3).

Whole genome sequencing (WGS) based genetic surveillance is critical for tracking and forecasting pathogen evolution. Phylogenetic analyses can estimate the spatiotemporal mobility of pathogens between communities around the world over the course of a pandemic. This has been essential for identifying a circulating Variant Being Monitored (VBM), Variant of Concern (VoC), or Variant of Interest (VoI) of SARS-CoV-2, the causative agent of coronavirus disease 2019 (COVID-19). Since the emergence of SARS-CoV-2 in late 2019, sustained transmission among the human host has generated numerous variants with specific phenotypic attributes. Mutations that potentially confer increased transmission, pathogenicity (4), immune escape, or resistance to therapeutics can be identified when sequenced and interpreted by combining *in silico* inferences with *in vitro* functional, animal, and epidemiological studies to link genotypes to phenotypes. Without genomic surveillance, some mutations may go unnoticed if they do not impact diagnostic assays.

Polymerase chain reaction (PCR) assays are susceptible to false negatives that result from mutations that weaken primer annealing. For example, a mutation or mutations causing one primer set to fail within a multiplex assay—called a partial assay failure—is best exemplified by the Alpha (B.1.1.7) variant. This variant was discovered first in the UK in December, 2020 by the so-called S gene target failure (SGTF) (aka the S gene dropout) in a Thermofisher TaqPath PCR test kit that targets 3 different regions (ORF1ab, E gene, and S gene) of the viral genome (5). A 6-nt deletion (Δ69–70 aa) resulted in the SGTF while the other two targets of the kit were positive for a given sample (5). After analyses determined the cause of the target failure was a new mutation, SGTF was used as a proxy for the presence of Alpha variant (B.1.1.7) in test samples. There are other examples of less severe assay failures in some commercial SARS-CoV-2 PCR tests (6, 7). Such failures affect biosurveillance network responses and public health policy decisions for disease control, containment, and prevention as they form the basis for our understanding of how an outbreak is progressing. Thus, systematic and periodic assessment of real-time PCR assay performance is critical for maintaining assay specificity and decreasing false negatives (6–10).

The Global Initiative on Sharing Avian Influenza Data (GISAID) organization and Nextstrain project provide genomic data, metadata, and phylodynamic analysis for monitoring ongoing outbreaks (11, 12). Continuous rapid sampling and sequencing of SARS-CoV-2 and sharing of data throughout the course of this pandemic provided a unique opportunity for visualizing signature erosion over time. Specifically, since January 20, 2020, we have used the PCR Signature Erosion Tool (PSET) to periodically evaluate assays *in silico* against all SARS-CoV-2 genome sequences from the GISAID EpiCoV™ database and share results on virological.org (13). PSET has been used previously to evaluate the impact of genomic drift on current and proposed assays for *Ebolavirus* and *Mammarenavirus* (14, 15).

Here, we present our assessment of diagnostic PCR assays *in silico* by applying PSET to regularly collected SARS-CoV-2 sequences. Recent alignment-based studies have been conducted with comparable results (8, 9). Our approach significantly increases the number of assays and subject sequences tested, breaks-down alignment rates over time by lineage, compares the mutation rate of assay target regions to the rest of the genome, and calculates the number of sequences producing false negatives for multiple assays.

## Materials and Methods

### PSET Analysis

**Figure 1:**
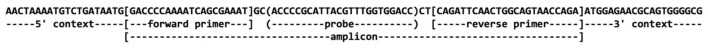
PSET assay definition. The assay definition includes square brackets and parentheses to delimit the primer sequences. Note that a probe is optional and that any sequence outside of the amplicon region is considered context for alignment purposes. This example corresponds to the cdc_n1 assay.

In this study we tested the *in silico* performance of 43 PCR assays (Supplementary Table 1) with different gene targets [19 ORF1ab, 8 spike (S) protein, 1 ORF3a, 3 envelope (E) protein, 1 ORF8, and 11 nucleocapsid (N)] against a set of 1,690,689 SARS-CoV-2 accessions (Supplementary Table 2) and sequences downloaded from the GISAID EpiCoV™ database on July 7, 2021 (11, 13, 16–26). At the time of analysis, the CDC listed Pango lineages P.1, B.1.351, B.1.1.7, B.1.427, and B.1.429 as VoCs and lineages P.2, B.1.525, and B.1.526 as VoIs (27, 28). Additional follow-up analysis was performed on the B.1.1.529 VoC as it emerged later. The PSET definition for each assay target was based on a reference amplicon sequence with delimited primer and probe regions (Figure 1). Twenty nucleotides of additional sequence context outside the amplicon were also included to inform alignment at the 5′ and 3′ ends. Context and inter-primer regions were obtained via global-local alignment of the assay primers to the SARS-CoV-2 reference genome by running the **glsearch36** program of the FASTA suite (29).

The first phase of PSET analysis queries the assay target definition against a BLAST+ database to search for matching subjects based on local alignment with the **blastn** program (30). Expansion of ambiguous DNA codes of the query is required for compatibility. For example, “GAWTAYA” has two, representing four expansions: “GAATACA,” “GATTACA,” “GAATATA,” and “GATTATA.” Only the first permutation is queried. An additional step re-evaluates the BLAST+ identity statistics by replacing the expanded query with the original one to account for ambiguous base similarity. Subjects with ≥85% identity to the amplicon region are then extended to cover the query range and extracted.

The second phase queries the corresponding primers separately against the library of extracted sequences to search for matching subjects based on global-local alignment with the **glsearch36** program, which is compatible with ambiguous DNA codes. Subjects with ≥90% identity are kept and aggregated by subject accession. A true positive (TP) is called if all primers aligned to the subject with the required identity and strand arrangement such that the primer would hypothetically amplify the target. The special case of a perfect true positive (PT) is called when there is 100% identity. Otherwise, a false negative (FN) is called. If the subject contains an N in any of the primer alignment regions, an N is appended to the category name (TPN and FNN). Doing so avoids removing low-quality, yet potentially informative sequences. An additional unknown (UNK) category is included to account for subjects that failed alignment during the first phase. In the case of near-neighbor analysis, a false positive (FP) or true negative (TN) is called when the taxonomy of the subject differs from the assay target. FPs with 100% identity are perfect false positives (PF).

Results were filtered to include high-coverage, human-host sequences with assigned Centers for Disease Control (CDC) and Pango lineages corresponding to VoC [Alpha (B.1.1.7), Beta (B.1.351), Gamma (P.1), Delta (B.1.617), Epsilon (B.1.427/B.1.429)] and VoI [Eta (B1.525), Theta (P.2), and Iota (B.1.526)] (27, 28). Metadata also included VoC and VoI designation times from disease control centers (31–39). Further aggregation and visualization was performed using the R programming language with the tidyverse (v1.3.1), lubridate (1.8.0), tsibble (v1.1.0), and cowplot (v1.1.1) packages (40–43). The PSET workflow itself was implemented using Biopython and Snakemake (44, 45). A summary of results and methodology refinements were posted to virological.org on a near-weekly basis (13).

### Variation Analysis

For variant analysis, the local GISAID EpiCoV™ database used for the PSET analysis was filtered to include only unambiguous DNA codes, resulting in a subset of 961,051 sequences. Single nucleotide variations (SNVs) and insertions or deletions (indels) were calculated by running the **nucmer** and **show-snps** programs of the MUMmer4 suite (46). Parameters for **nucmer** included NCBI GenBank accession no. NC_045512.2 (47) as the reference and flags to search the forward strand of each query (-f) with 28 threads (-t). The **show-snps** program transformed the resulting delta file into a tabular file (-T) of SNP/indel calls. An R script loaded the variants identified via MUMmer4 and split the data into two groups according to overlap with assay primer and probe target regions. The percentage of positions with ≥n mutations was calculated, where n was in the range [0, 500]. A two-sample Kolmogorov-Smirnov procedure tested the null hypothesis that the observed mutation percentages arose from the same distribution for the assay target and non-target genomic regions.

### Omicron Analysis

The Omicron (B.1.1.529) wave emerged after the initial analysis was completed. Accordingly, an updated analysis targeted this VoC. On February 16, 2022, genomes were downloaded manually from the EpiCoV™ search page. Filters were enabled to include complete sequences with high coverage and collection dates. The PSET workflow analyzed the final set of 48,358 sequences (Supplementary Table 3) as previously described.

## Results

### Assay Regions and Genomic Variation

**Figure 2:**
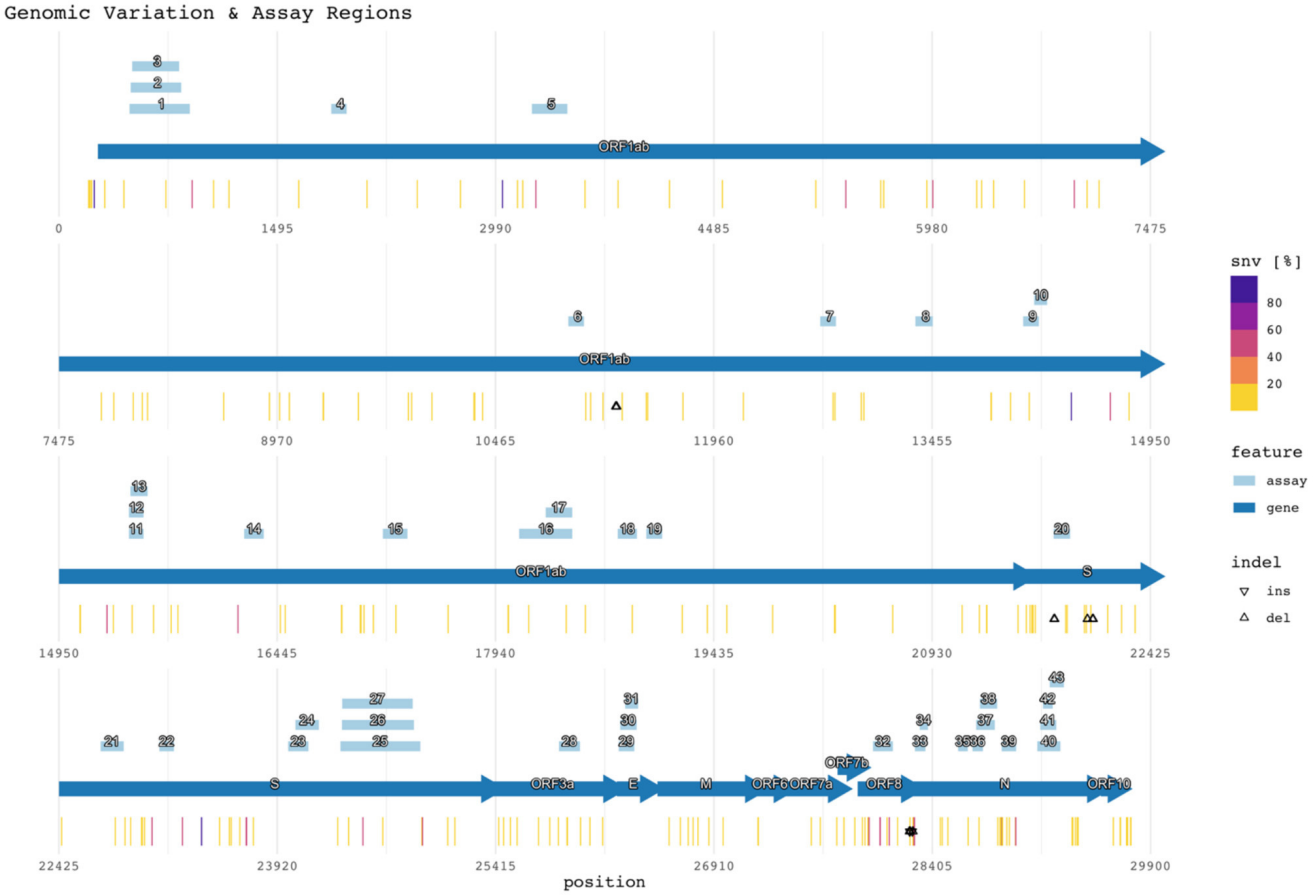
Genomic variation and assay regions. The numbered rectangles and labeled arrows indicate PCR assay and gene regions respectively. Heat values indicate the percentage of SNVs observed at the reference location across all subjects. Percentages for both SNVs and indels are compiled in Supplementary Table 4.

Figure 2 shows the target region of each assay with respect to the reference SARS-CoV-2 genome (NCBI GenBank accession no. NC_045512.2), SNVs, and indels (47). Mutations with <1% prevalence (~99.62% of all positions with observed mutations) were excluded from the figure but remain listed in Supplementary Table 4. The figure also plots the assay target regions on the genome. Plotting all the regions together with respect to mutations helps visualize signature erosion due to genetic drift.

**Figure 3:**
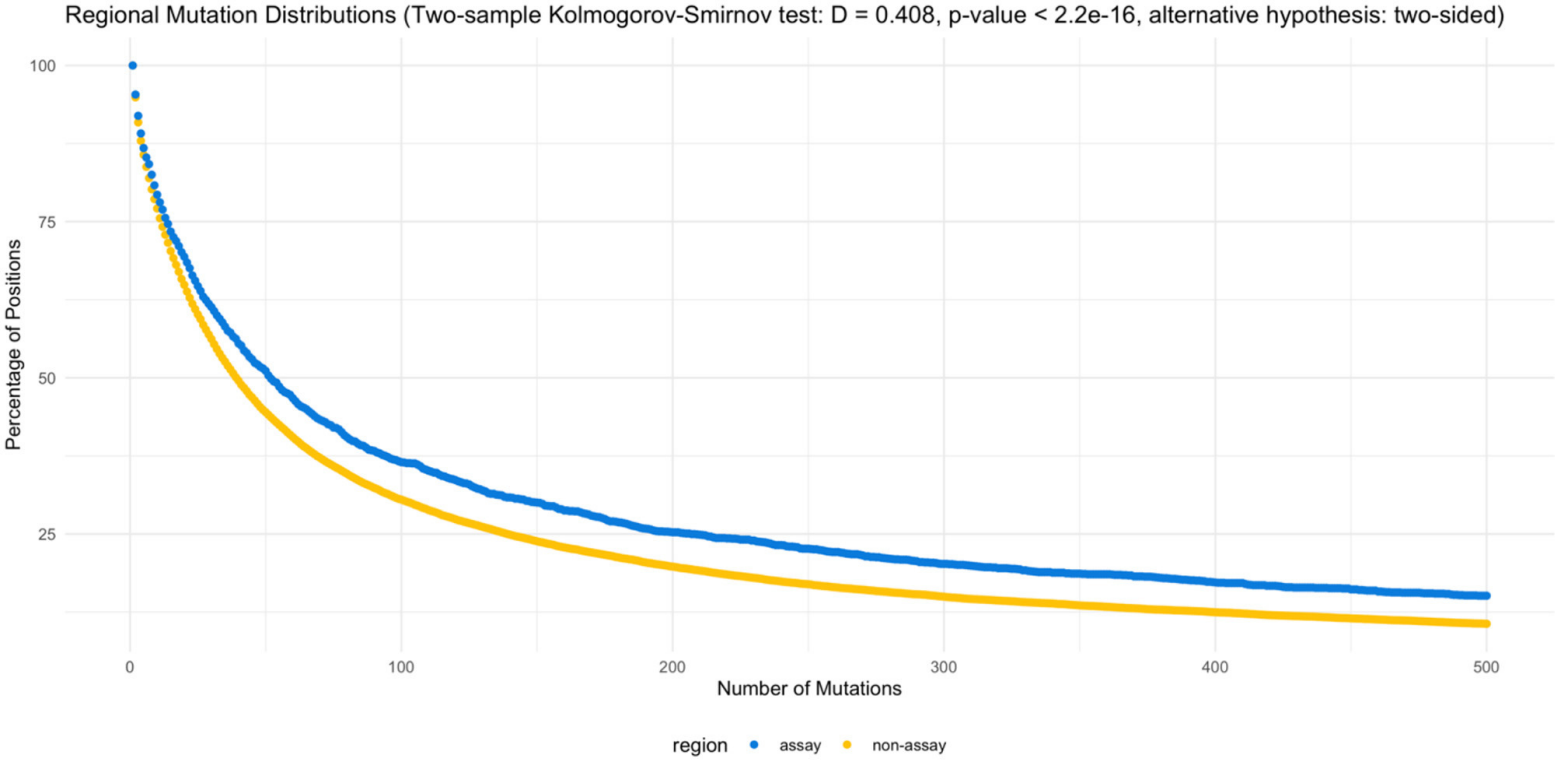
Regional mutation distributions. The two-sample Kolmogorov-Smirnov test rejected the null hypothesis (α = 0.05), supporting the elevated number of mutations observed in the assay regions (D = 0.408, *p* < 2.2e−16, alternative hypothesis: two-sided). Supplementary Tables 5, 6 list the counts by position and percentage of mutations by region.

On the other hand, when the assay target regions are compared specifically against the non-target regions, assay target regions with mutations are present in higher percentage of genomes (Figure 3). The two-sample Kolmogorov-Smirnov test obtained a *p* value of 2.2E−16, rejecting the null hypothesis at a significance level of 0.05 in favor of the two-sided alternative.

### Assay Alignment Confusion Matrix

The assays were able to correctly detect the vast majority of the 1,690,689 genome sequences according to the *in silico* PSET analysis. Of the 43 assays, 34 aligned with 100% identity to over 1.6 million subjects. Otherwise, TP rates exceeded 98%, except for the Young-S and China_N assays, which exhibited high FN rates at ~47% and ~59% respectively. The TP percentage was calculated as the sum of PT, TP, and TPN divided by the total number of subjects. Alignments exceeded identity threshold for most corresponding subject amplicon sequences, only failing on average ~0.74% (UNK%) of the time. Table 1 and Supplementary Table 7 include a full breakdown by assay. In the separate Omicron study, most of the assays (40 / 43) were able to detect sequences with a TP rate of ≥96%, with Young_S, Thailand_WH-NIC_N, and China_N assays having reached TP rates of 75.64%, 2.98%, and 0.34% respectively (Supplementary Table 8). We also looked at the specificity of the assays against near-neighbor sequences (Supplementary Table 9), where 8 showed significant false positives (FP) and perfect FPs (PFs) (those with 100% identity).

**Table 1:** Confusion matrix of assay calls based on alignment/arrangement.

**ID**	**Assay**	**From**	**To**	**Gene**	**PT**	**TP**	**TPN**	**FN**	**FNN**	**UNK**
1	Japan_NIID_WH-1_F501	483	896	ORF1ab	1,657,346	20,369	178	89	116	12,591
2	Japan_NIID_WH-1_F509	491	837	ORF1ab	1,640,106	37,112	429	201	268	12,573
3	Japan_NIID_WH-1_Seq_F519	501	823	ORF1ab	1,634,109	28,526	433	6,616	8,419	12,586
4	Yip-ORF1ab	1,865	1,970	ORF1ab	1,661,320	16,261	408	18	432	12,250
5	Noblis.12	3,239	3,482	ORF1ab	1,644,652	33,339	283	241	69	12,105
6	C1_COV_ORF1a	10,964	11,071	ORF1ab	1,643,241	27,716	2,061	4	293	17,374
7	France_nCoV_IP2	12,689	12,797	ORF1ab	1,647,399	30,804	280	125	59	12,022
8	China_ORF1ab	13,341	13,460	ORF1ab	1,664,710	13,037	394	18	255	12,275
9	France_nCoV_IP4	14,079	14,186	ORF1ab	1,554,134	124,086	300	349	39	11,781
10	Young-ORF1ab	14,154	14,243	ORF1ab	1,653,742	24,466	238	111	299	11,833
11	ncov_rdrp_1	15,430	15,530	ORF1ab	0	1,676,313	179	792	1,624	11,781
12	ncov_rdrp_2	15,430	15,530	ORF1ab	71	1,677,034	238	1	1,564	11,781
13	Won-ORF1ab	15,440	15,558	ORF1ab	1,563,669	114,920	202	2	36	11,860
14	Chan-ORF1ab	16,219	16,353	ORF1ab	57	1,677,405	254	72	250	12,651
15	Noblis.40	17,169	17,337	ORF1ab	1,617,536	61,076	181	110	63	11,723
16	Noblis.44	18,102	18,466	ORF1ab	1,660,926	17,339	268	68	41	12,047
17	Noblis.42	18,284	18,466	ORF1ab	1,661,715	16,697	263	22	60	11,932
18	HKU-ORF1b-nsp14	18,777	18,909	ORF1ab	1,663,433	15,101	226	81	30	11,818
19	C2_COV_ORF1b	18,973	19,082	ORF1ab	1,632,389	45,440	893	1	87	11,879
20	Young-S	21,762	21,876	S	775,271	62,150	2,900	798,480	13,942	37,946
21	Chan-S	22,711	22,869	S	1,607,062	73,732	1,317	109	1,540	6,929
22	Won-S	23,113	23,213	S	1,666,248	12,686	808	849	2,221	7,877
23	C5_COV_S_gene	23,995	24,134	S	1,657,631	24,567	595	40	216	7,640
24	Noblis.57	24,045	24,205	S	1,655,637	26,634	406	112	136	7,764
25	Japan_WuhanCoV-spk1	24,353	24,900	S	1,664,354	17,288	234	302	86	8,425
26	Japan_NIID_WH-1_F24381	24,363	24,856	S	1,656,823	24,516	512	349	76	8,413
27	Japan_NIID_WH-1_Seq_F24383	24,365	24,848	S	1,655,758	22,709	467	3,235	109	8,411
28	C3_COV_ORF3a	25,849	25,993	ORF3a	1,513,748	160,268	1,757	48	751	14,117
29	Won-E	26,258	26,365	E	5	1,678,087	110	74	300	12,113
30	ncov_e_gene	26,268	26,381	E	1,671,863	6,099	139	44	288	12,256
31	Niu-E	26,302	26,391	E	1,661,887	15,870	341	26	264	12,301
32	C4_COV_ORF8	27,999	28,135	ORF8	833,817	828,366	9,457	2,861	388	15,800
33	cdc_n1	28,286	28,358	N	1,621,043	56,882	407	29	48	12,280
34	Thailand_WH-NIC_N	28,319	28,376	N	1,649,541	28,495	242	101	49	12,261
35	Young-N	28,582	28,648	N	1	1,677,953	153	33	138	12,411
36	cdc_n3	28,680	28,752	N	1,614,506	63,680	429	10	39	12,025
37	ncov_n_gene	28,705	28,833	N	1,633,141	42,891	399	218	153	13,887
38	Won-N	28,731	28,849	N	1,634,741	41,592	217	114	34	13,991
39	China_N	28,880	28,979	N	401,644	258,102	569	1,010,632	6,168	13,574
40	Japan_NIID_2019-nCOV_N	29,124	29,282	N	3	1,676,346	196	200	177	13,767
41	HKU-N	29,144	29,254	N	1,633,061	43,092	608	196	190	13,542
42	cdc_n2	29,163	29,230	N	1,622,617	53,899	565	79	130	13,399
43	Chan-N	29,209	29,306	N	1,630,595	45,578	368	65	223	13,860

*True positive (TP); false negative (FN); perfect TP (PT); TP/FN aligned with N's (TPN/FNN); unknown (UNK)*.

### Assay Alignment Identity Over Time

**Figure 4:**
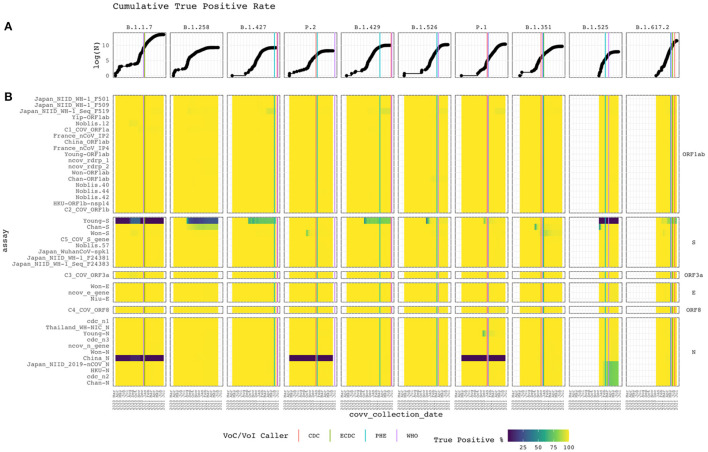
Cumulative true positive rate. Vertical and horizontal facets divide the graph by Pango lineage and assay target gene. **(A)** The top line graph shows the logarithm of the cumulative total of subject sequences. **(B)** Heat map of the current PCR assays with the cumulative conditional true positive rate assessed from Apr 2020 to Feb 2021. The assay targeting specific genes are labeled on the right. White represents the absence of subject sequences of the lineage in the reference database. Vertical lines when each disease control organization called the lineage a VoC/VoI based on available data (compiled in Supplementary Table A).

Alignment identity of all assays against sequences of different lineages of SARS-CoV-2 was assessed from 2020-03-15 to 2021-07-05. Figure 4 depicts heat maps of the TP rate over time for each VoC/VoI with a corresponding line graph of the cumulative log-total number of sequences. Only GISAID sequences with collection date metadata specifying year, month, and day were included in the heatmap. The graph reveals sudden changes in assay alignment identity with respect to variant abundance.

Alignment identity remained constant for most assays, with perfect or near-perfect TP rates. However, some interesting patterns were observed. Some assays targeting the S and N genes performed poorly. For example, Young-S exhibited low TP rates within the B.1.1.7 and B.1.525 lineages and faltered for B.1.258, B.1.427, and B.1.429 isolates as cumulative sampling increased. Other assays targeting the S protein gene performed well, despite some temporary rough patches for Chan-S, Won-S and C5_COV_S_gene. Recently, Chan-S appears to have started failing for the B.1.258 lineage. China_N failed for the B.1.1.7, P.2, and P.1 clades. All other assays targeting the N gene performed well. However, Japan_NIID_2019-nCOV_N, HKU-N, cdc_n2, and Chan-N recently started failing for the B.1.525 lineage. Also, Young-N temporarily exhibited a low TP rate before recovering.

### VoC/VoI Call Patterns

Figure 4 displays the timeline for each lineage to be identified as a VoC/VoI by the CDC, European CDC (ECDC), Public Health England (PHE), and World Health Organization (WHO). Most identifications occurred within a month of each other. However, for P.2 and B.1.429, 186 and 118 days passed between the initial and final call. The CDC and PHE called the former within 12 days of each other and the former within 111 days. In both cases, the WHO called last. We found no evidence of a call from the ECDC in these cases. The B.1.429 case shows the initial call occurring during a period of low alignment identity for the Young-S assay and the beginning of a decline for the Japan_NIID_WF-1_Seq_F519 assay. Despite the varying times at which these lineages were identified, when looking at the cumulative increase of the GISAID sequences for each, it is readily apparent that they initially occurred at very low proportions. This increase, and the resulting VoC/VoI designations, underscore the importance of continual monitoring from multiple organizations offering different perspectives and criteria with respect to spatiotemporal trends.

### *In silico* False Negative Distribution by Assay

As assay targets were distributed throughout the genome, we were also interested in seeing whether emerging variants possessed mutations that resulted in more than one assay producing a predicted FN. Supplementary Table SB shows that nearly all (>99%) sequences resulted in a predicted true positive in at least 41 of 43 assays. Also, Supplementary Table SC shows that the majority (97%) of sequences producing a single FN were observed to originate from the China_N (87%) or Young-S (10%) assays. The majority (765,813) of sequences produced FNs in two different assays, China_N and Young-S, while 643,876 sequences did not produce an FN in any of the 43 assays. We observed a substantial drop-off in the number of sequences which caused FNs in 3 or more assays. This suggests that it is unlikely for a sequence to produce FNs in more than 2 assays and that a multiplex panel with any of the other 41 assays evaluated here would likely perform successfully.

## Discussion

### Signature Erosion Analysis

The rapid increase of novel SARS-CoV-2 variants raises concerns regarding the efficacy of PCR-based diagnostic assays. This study assesses the potential signature erosion of the current COVID-19 real-time diagnostic assays using the GISAID sequence database. We find that 41 out of the 43 assays continue to perform very well throughout the observed timeframe even as new variants arose. Additional analysis completed after the emergence of Omicron revealed that 40 of 43 assays continued to perform well.

Variants containing mutations within the target regions for primer and probe hybridization were observed to have a substantial effect on predicted assay alignment identity. The characteristic S:Δ69/70 mutation of the Alpha variant occurs within the forward primer of the Young-S assay target, while mutations N:R203K and N:G204R occurred within the borders of the China_N assay target, resulting in PSET calling significant FN rates of those assays. The China_N assay failed completely for the lineages B.1.1.7, P1, and P2. This may be due to a “GGG” to “AAC” mutation in the forward primer, as observed in previous studies (8, 9). The 3-nt substitution mutation could potentially reduce the China_N forward primer binding affinity, which could significantly increase the FN rate, especially since it occurs at the 5′-end. The B.1.1.529 lineage contains an N:Δ31/33 deletion (48) which falls inside the borders of the Thailand_WH-NIC_N assay and results in PSET predicting significant FN rates. This may be a similar situation as described above where primer binding affinity is affected by this type of mutation.

Various point mutations at the primer binding motifs are observed in all the lineages, which did not affect overall performance according to evaluation of *in silico* alignments. The exception was for lineage B.1.258, which bears a complementary sequence to the China_N primer resulting in perfect alignment. The China_N and Young-S assays experience 56 × and 44 × the FN rate of all other assays combined, respectively. In almost all assays, there is a percentage of mutations in one of the targets of the assay.

### Recent Studies and Future Direction

The COVID-19 pandemic has demonstrated the need for the *in silico* monitoring of PCR-based diagnostic assay performance. Furthermore, given that surveillance network data are widely available to aid in quick-turnaround research and development of replacement assays, *in silico* monitoring has become an important early warning indicator (10). Our approach is consistent with recent alignment-based studies by confirming the overall high assay target sequence identity and detection of the China_N mutation (8, 9). We also observed potential early signs of signature erosion prior to official variant calls. Future research can further elucidate validation of *in vitro* and *in silico* predictions while considering the clinical and public health relevance. For example, the PSET algorithm could incorporate mismatch position, since mismatches near the 3′ end of the probe sequence potentially have a greater impact on assay performance (49, 50). Additional wet lab experiments can help systematically select alignment thresholds and motivate algorithmic refinements.

## Conclusion

This study highlights the consequences of mutations in SARS-CoV-2 genomes on PCR-based diagnostic assays. The importance of real-time monitoring of molecular assay alignment *in silico* is highlighted by the discovery of the Alpha variant. The failure of one primer/probe combination within the multiplex assay targeting a region of a deletion in this variant underscored the need for extensive sequence-based surveillance. Sustained transmission and proliferation of viruses such as SARS-CoV-2 in a global pandemic leads to rapid evolution and accumulation of mutations that confer advantageous phenotypes, such as potentially evading diagnostics, therapeutics, and vaccines. Real-time *in silico* monitoring of assay signature erosion allows for the redesign and refinement of diagnostic assays to address assay and medical countermeasure failures to avoid dire failures of medical countermeasures.

## Data Availability Statement

The original contributions presented in the study are included in the article/Supplementary Material, further inquiries can be directed to the corresponding author/s.

## Author Contributions

SS: conceptualization and supervision. DN, JK, and SW: data curation. DN, JK, SM, and SW: formal analysis. BG: funding acquisition. DN, JK, SM, LB, and SS: investigation. DN, JK, SM, MH, SW, and SS: methodology. BG and SS: project administration. DN, MH, and SW: software. DN, JK, SM, SW, JV, and SS: validation. DN and SW: visualization. DN, JK, SM, and SS: writing—original draft. DN, JK, SM, JV, LB, KJ, SG, and SS: writing—review and editing. All authors contributed to the article and approved the submitted version. The following CRediT (Contributor Roles Taxonomy) terms indicate the corresponding initials of the contributing authors.

## Funding

Funding for this work was provided by Joint Program Executive Office, JPL-CBRND-EB, DBPAO under the contract number W911QY-17-C-0016.

## Conflict of Interest

DN, JK, SM, MH, SW, LB, and KJ were employed by Noblis, Inc. SG was employed by Noblis ESI. SS was employed by Logistics Management Institute. The remaining authors declare that the research was conducted in the absence of any commercial or financial relationships that could be construed as a potential conflict of interest.

## Publisher's Note

All claims expressed in this article are solely those of the authors and do not necessarily represent those of their affiliated organizations, or those of the publisher, the editors and the reviewers. Any product that may be evaluated in this article, or claim that may be made by its manufacturer, is not guaranteed or endorsed by the publisher.
